# Age-related differences in inhibitory control and memory updating in boys with Asperger syndrome

**DOI:** 10.1007/s00406-016-0756-8

**Published:** 2016-12-26

**Authors:** Elisabeth M. Weiss, Bianca Gschaidbauer, Liane Kaufmann, Andreas Fink, Günter Schulter, Erich Mittenecker, Ilona Papousek

**Affiliations:** 1 Biological Psychology Unit, Department of Psychology, University of Graz, Univ.-Platz 2, 8010 Graz, Austria; 2 Department of Psychiatry and Psychotherapy A, General Hospital Hall, Tirol, Austria

**Keywords:** Autism spectrum disorder, Mittenecker pointing test, Cognitive flexibility, Inhibition, Memory updating

## Abstract

Deficits in specific executive domains are highly prevalent in autism spectrum disorder; however, age-related improvements in executive functions (reflecting prefrontal maturational changes) have been reported even in individuals diagnosed with autism. The current study examined two components of cognitive flexibility (inhibition of prepotent responses and memory monitoring/updating) by using a random-motor-generation task (MPT) in a group of 23 boys with Asperger syndrome (AS) and 23 matched healthy controls. We found poorer inhibition and more repetitive responses in younger AS children solely, but comparable memory monitoring/updating skills across groups. Overall, our findings correspond well with previous studies and reveal that even in AS specific EFs may improve with age and, thus, call for a more differentiated view of executive (dys) function profiles in children diagnosed with AS. Tests such as the random-motor-generation task may help to disentangle more specific processes of executive deficits in autism spectrum disorder as compared to the more classical tests.

## Introduction

Autism spectrum disorder (ASD) is a pervasive developmental disorder characterized by difficulties in social interaction and communication as well as restricted and repetitive behaviors [1]. Furthermore, deficits in specific executive domains are highly prevalent in individuals diagnosed with autism, having even been considered to be neuropsychological core deficits of autism [e.g., 2–6]. Executive functions (EFs) are an umbrella term and include higher-level cognitive processes such as planning, mental flexibility, inhibitory control and working memory. EFs are primarily mediated by the prefrontal cortices that are known to show a protracted developmental trajectory into late adolescence and even early adulthood in both healthy individuals (e.g., [7, 8]; for recent brain imaging findings, see [9, 10]) and individuals with autism (for a review, see [11]). Despite converging evidence revealing that various EFs (e.g., planning, cognitive flexibility or working memory) are impaired in individuals with autism (for a recent review see [12]), to the present no specific EF patterns unique to individuals diagnosed with autism have been identified [13–15]. Moreover, long-term follow-up studies disclosed high intra- and interindividual variability in EFs growth trajectories in children diagnosed with autism (for a review, see [16]).

Asperger syndrome (AS) is considered to be among the milder forms of autism (Diagnostic and statistical manual of mental disorders (4th ed.; DSM-IV; [17]), and 10th revision of the International Statistical Classification of Diseases and Related Health Problems (ICD-10; World Health Organization (WHO); [18]); for a critical review, see [19]); however, in the new Diagnostic and Statistical Manual of Mental Disorders (5th ed.; DSM-5; [1]), the specific AS diagnosis has been removed and now there is only the diagnosis of ASD. While social-communication difficulties and restricted patterns of interest and behavior are key symptoms of AS (that are shared with other forms of autism), individuals with AS—unlike those with other forms of autism—do not present with significant language delays and generally exhibit average overall cognitive abilities. The evidence of executive dysfunctions in AS is equivocal, with some studies showing no EF deficiencies (children and adolescents: [20, 21]; adults: [22, 23]), while others found marked executive deficits in children and adults alike [13, 24–27]. Some authors suggest that EF deficits in adults with AS and high-functioning autism may not be apparent in standard neuropsychological tests of EFs such as the Wisconsin card sorting test (WCST) which are rather unspecific indicators of brain functions because they confound several cognitive components and processes [2, 28, 29]. Therefore, more sensitive neuropsychological tools enabling researchers to parse and segregate the cognitive processes of interest are warranted.

Miyake et al. [30] describe three key aspects of EFs consisting of “shifting,” “updating” and “inhibiting prepotent responses.” Shifting involves cognitive flexibility, which refers to the ability to dynamically activate and modify cognitive processes in response to changing conditions and demands. Inhibition refers to the ability to inhibit or override the tendency to produce a dominant or prepotent response necessary to achieve a current behavioral goal. Finally, updating refers to the ability to monitor incoming information and to adjust the content of working memory according to the current behavioral goal [30]. Notably, inhibition and (working) memory updating are considered to be building blocks that develop before more complex EFs such as cognitive flexibility [31]. In the reminder of this work, we will focus on the aforementioned key aspects of EFs as described by Miyake and collaborators [30].

Random generation tasks require participants to generate a random sequence of items. For instance, in the random number generation task, the most popular variant, participants are instructed to produce long sequences of numerical digits (mostly 1–9) “as random as possible,” mostly in synchrony with a pacing stimulus for a number of trials. In “pure” random generation tasks, no additional instruction is given, whereas in “pseudorandom” generation tasks, instructions like “avoid repetitions, number patterns” are included. For successful task performance in random number generation, the participant has to continuously select a new response from a set of possible alternatives, memorize this set of response alternatives, suppress prepotent response patterns such as repetitions and counting, and monitor and change response production [32, 33]. Previous behavioral and imaging studies have shown that random generation tasks are related to executive processes and the capacity of working memory and that especially the (pre)frontal lobes are playing a critical role in the monitoring of habitual responses [33–37]. Random response generation tasks have been proven to be useful diagnostic tools for the identification of clinically relevant impairments of EFs in psychiatric and neurological disorders such as schizophrenia [38–42] and Parkinson’s disease [43–45]. Moreover, random response generation tasks were used to examine inhibitory control in individuals diagnosed with autism. Upon using a pseudorandom number generation task, Williams et al. [46] showed that low-functioning individuals with autism were more likely to repeat previous digits in comparison with IQ- and age-matched controls (thus reflecting inhibition deficits). Similarly, upon using a verbal equivalent of the pseudorandom generation task, Rinehart et al. [47] found that compared to controls, children with high-functioning autism repeated single numbers more frequently than control children, while children diagnosed with AS generated more repetitive number patterns. Furthermore, Rinehart and colleagues [47] reported that individuals with AS (but not controls) did benefit from external auditory cueing (i.e., children with AS produced fewer repetitive number patterns under the cued compared to the uncued task condition). Consequently, Rinehart et al. [47] proposed that while external cueing might have aided the inhibition of prepotent response tendencies in individuals with AS, external cueing seemed to have a distracting effect on healthy controls. Importantly, the random generation tasks used by Rinehart et al. [47] and Williams et al. [46] required participating individuals with autism to randomly generate numbers and, thus, probably provoked confounds with (more or less) overlearned counting routines. Furthermore, the random number generation tasks used in the latter studies examined inhibitory processes solely (by assessing individuals’ ability to inhibit repetitive response tendencies).

A main aim of the present study was to tease apart cognitive (sub)processes underlying deficits in cognitive flexibility in individuals diagnosed with AS [5, 19, 48]. Previously, executive dysfunctions such as poor regulation and inhibitory control of behavior or lack of flexibility have been linked to repetitive and stereotyped behavior (for a review see [49]). To explain repetitive and stereotyped behavior in children with autism, Turner [50] proposed two separate hypotheses, one relating to an inability to inhibit prepotent responses and another related to an inability to “spontaneously generate novel behavior without prompting.” However, until now several studies could not fully substantiate either hypothesis mainly because study results concerning executive impairments in AS are highly variable which might be due to methodological heterogeneities between studies including type of assessment tests used, child age, overall cognitive ability, and language skills significantly modifying results in assessment tasks (for a review see [49].

Hence, we used a motor version of the random generation task (the so-called Mittenecker pointing test/MPT) that enabled us to separately measure two components of cognitive flexibility mentioned above [30, 51], namely the inhibition of prepotent responses (i.e., the inhibition of developing routines) as well as memory monitoring and updating by use of sophisticated and validated indexes derived from the produced “random” sequences of chosen keys. Most importantly, unlike random number (or letter) generation tasks, the MPT does not require the suppression of overlearned response sequences (such as counting up or down or producing letters in alphabetical order). Hence, the MPT does not draw on academic skills such as counting or spelling that may vary considerably across participants. In addition, unlike random number or letter generation tasks, the MPT does not require memorizing the set of response alternatives. Thus, it allows more straightforward interpretation. To our knowledge, this is the first study in the field of autism utilizing a random generation task that is based on motor responses that are neither overlearned nor confounded with academic skills such as counting or spelling.

A further goal of the present study was to examine whether the aforementioned aspects of cognitive flexibility are subjected to age-related changes (i.e., improvements) in our study group comprising children and adolescents diagnosed with AS. Geurts et al. [52] showed in their meta-analysis that age moderated the performance on prepotent response inhibition tasks with younger individuals diagnosed with autism exhibiting poorer response inhibition in tasks such as the Go/No-Go test or the Stop signal test.

We hypothesized that poorer inhibition and more repetitive response patterns will be only evident in young children diagnosed with AS (thus reflecting age-related improvements of response inhibition in adolescents diagnosed with AS). We expected differences to appear primarily in the inhibition of developing routine response patterns, which was hypothesized to be connected to higher-level repetitive behaviors in autism (cf. Rinehart et al. [47]), and did not expect marked differences in the memory monitoring and updating component.

## Materials and methods

### Participants and procedure

Twenty-four boys with AS (age range 5.7–14.3 years) were recruited from a consulting center for individuals with autism and AS in Graz, Austria. Diagnostic criteria of AS conformed to ICD-10 (F84.5; DIMDI [53]), as diagnosed by a child psychiatrist. Twenty-four typically developing boys (TD), matched for age and overall intellectual functions (using the culture fair intelligence test), were recruited as controls.

Two children (one AS and one TD) of the younger age group were excluded from the analyses because they were not able to comply with task instructions. Thus, the final sample comprised 23 boys with AS (*M* = 10.1 ± 2.7 years old) and 23 TD boys (*M* = 10.0 ± 2.8 years old; *t*(44) = 0.12, *p* = .90, *η*_*p*_^2^ = .00). Ten boys of the AS group (none of the TD group) had an additional diagnosis of attention disorder and were treated with Ritalin, Atomoxetine, or atypical neuroleptica (Risperidone, Olanzapine).

Participants were tested individually. They were introduced to the experimental task (i.e., MPT) by a child psychologist and were given some practice trials to ensure task comprehension. In a separate test session, the age-appropriate form of the German standardization of the culture fair intelligence test (CFT) was administered to obtain a current estimate of nonverbal intelligence of all participants (CFT1 [54]; CFT 20-R [55]). Importantly, the two diagnostic groups did not differ regarding their intelligence scores (AS *M* = 113.5 ± 10.3, TD *M* = 114.1 ± 11.1; *t*(44) = 0.18, *p* = .86, *η*_*p*_^2^ = .00).

The study was in accordance with the 1964 Declaration of Helsinki and was approved by the local Ethics Committee. Informed written consent was obtained from parents of all children and adolescents prior to participation.

### Mittenecker pointing test (MPT)

The MPT is a computer-based test requiring participants to press (with their index finger) the keys of a keyboard with nine unlabeled keys irregularly distributed over the board in the most random or chaotic order possible (for more details concerning the task please see [51, 56]). The responses were paced by an acoustic signal (1.2/s.) to control the rate of production. A total of 180 responses were required. The instruction was: “the task you have to accomplish is very easy and simple. Here is a set of nine black keys, all equal. Your task is to press the various keys in a completely random order. Most importantly, please do not stick to a certain sequence or order, but press the keys in an as random sequence as possible. Just select the succession of keys by mere chance. You will do it with the index finger of your right hand. Speed is not important, so do not hurry but try to follow the rhythm of the acoustic signal.” If the task was not clearly understood, further information was given to illustrate the concept of randomness, including phrases such as “lottery-like pressing.” A brief demonstration of 10 successive trials was given by the examiner, who produced a standardized, pseudorandom sequence, alternating small movements, large movements and repetitions on the same key. Then participants completed 10 practice trials to get accustomed to the acoustic pace, before the actual test was started.

As outcome variables, we used two quantitative measures of deviation from randomness that are based on information theory analysis [40, 51, 57], namely symbol redundancy (SR) and context redundancy (CR). According to a key assumption proposed by information theory analysis, information is maximal when redundancy is minimal and the series approximates randomness (i.e., maximal disorder).

SR taps the memory component (memory monitoring/updating) of random sequence generation [30, 51] and refers to the inequality of the relative frequencies of chosen keys. A SR score of zero denotes maximal equality of the relative frequencies and, thus, minimal predictability, whereas a score of 1.0 denotes maximal redundancy and, thus, a complete lack of randomness. SR is equivalent to what in the literature is sometimes termed *R* score.

CR examines the inhibition of prepotent responses and is based on the sequential probability of each chosen key. In true random series, all possible dyads (pairs of adjacent responses) are approximately equiprobable, whereas their frequencies deviate from equality if responses are continuously influenced by previously chosen alternatives. The major part of the interindividual variance in CR is due to the tendency to repeat certain response sequences en bloc [40]. Hence, CR reflects the inhibition of developing routines [30]. A CR score of zero denotes the complete absence of any regular pattern, while a score of 1.0 denotes the presence of a fixed, repetitive response pattern (i.e., maximal perseveration). For detailed information on the test and how to compute SR and CR, see [51].

### Statistical analysis

The research question was tested with two analyses of variance, using diagnosis (AS vs. TD) as a dichotomous between-subjects variable and age as a covariate, i.e., continuous between-subjects variable. A significant interaction between the two independent variables indicates that age moderates the differences between AS and TD boys. One analysis was done with memory updating (SR), and one with inhibition of developing routines (CR) as the dependent variable. In supplementary analyses, it was tested whether the presence or absence of a comorbid attention disorder mediates the effects of interest within the experimental group (two independent *t* tests). Estimates of effect sizes are reported using partial eta-squared (*η*_*p*_^2^), which gives the proportion of variance a factor or interaction explains of the overall variance in the dependent variable. All statistical tests were performed with *α* = .05 (two-tailed).

## Results

No group differences were observed between children and adolescents with AS and TD children/adolescents in the SR score (diagnosis *F*(1,42) = 0.0, *p* = .94, *η*_*p*_^2^ = .00; interaction diagnosis × age *F*(1,42) = 0.1, *p* = .78, *η*_*p*_^2^ = .00; age *F*(1,42) = 0.0, *p* = 84, *η*_*p*_^2^ = .00; mean SR scores were .007 ± .003 in TD boys and .009 ± .006 in AS boys).

**Fig. 1:**
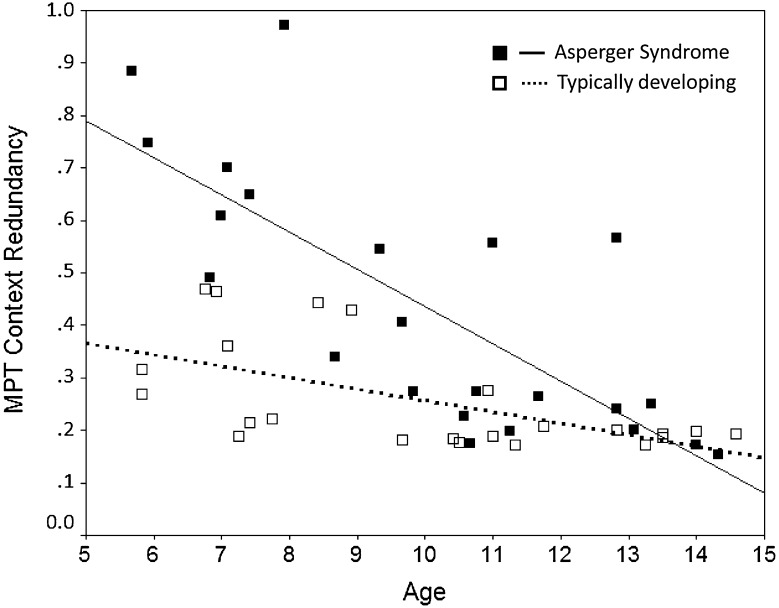
Moderating effect of age on differences between boys with Asperger syndrome and typically developing boys in the inhibition of developing routines (MPT context redundancy). *Note* Significant interaction effect of diagnosis by age. The regression lines show the estimated CR scores in years 5–15 for boys with AS and TD controls, respectively. Higher CR scores indicate poorer inhibition of developing routines

The analysis of CR revealed significant main effects of diagnosis (*F*(1,42) = 20.5, *p* < .001, *η*_*p*_^2^ = .33) and age (*F*(1,42) = 42.4, *p* < .001, *η*_*p*_^2^ = .50), but also an interaction of diagnosis by age (*F*(1,42) = 11.9, *p* < .001, *η*_*p*_^2^ = .22). Figure 1 shows the corresponding regression lines (estimated CR scores in years 5–15) for boys with AS and TD boys, respectively, as well as the raw scores for all participants. Mean CR scores were .26 ± .10 in TD boys and .43 ± .24 in AS boys. The results indicate poorer inhibition of prepotent responses in boys with AS than in TD boys, which was only evident in younger boys, while performances in older boys with AS were increasingly similar to TD controls (Fig. 1). Both regression lines were significant (AS *β* = −0.77, *p* < .001; TD *β* = −0.59, *p* = .003).

Importantly, boys with AS with (*n* = 10) and without (*n* = 13) comorbid attention disorder did not differ regarding their CR (*t*(21) = 1.4, *p* = .18, *η*_*p*_^2^ = .08; with: .35 ± .16, without: .49 ± .28) or SR scores (*t*(21) = 0.31, *p* = .76, *η*_*p*_^2^ = .00; with: .009 ± .005, without: .010 ± .007). Additionally, they did not differ regarding their age (*t*(21) = 1.3, *p* = .22, *η*_*p*_^2^ = .07; with: 10.9 ± 2.1, without: 9.5 ± .2.9) or IQ (*t*(21) = 1.2, *p* = .26 *η*_*p*_^2^ = .06; with: 110.7 ± 14.3, without: 115.7 ± 5.3).

## Discussion

It has been posited that some EFs are difficult to quantify experimentally, because they are rooted in the temporal domain [58]. This is particularly true for measures of cognitive flexibility, which refers to the function of dynamically activating and modifying cognitive processes in response to changing conditions and demands. The examination of the temporal organization of behavior requires a behavioral paradigm that enables the repeated measurement of many observations of behavior. The current study analyzed the sequential response patterns in a motor random generation task (MPT) in children with AS and matched healthy controls. Studies using random generation tasks in children and adolescents with autism have been sparse to date [46, 47], and previous studies required participants to suppress (more or less) overlearned counting or spelling routines. The MPT provides independent indicators of (1) inhibition of dominant or prepotent responses (outcome variable CR) and (2) memory monitoring/updating (outcome variable SR), both of which tap distinct components of cognitive flexibility. Taken together, the methodological approach of the present study significantly extended that of previous studies.

In our study, we found higher CR scores in boys with AS than in TD boys; however, this was only evident in younger boys, while performances in older boys with AS were rather similar to TD boys and approached CR scores typically observed in healthy adults [51]. CR reflects the extent to which responses are continuously influenced by previously chosen alternatives. Thus, CR may be regarded as indicator of the (in)efficiency of inhibitory processes, specifically of the inhibition of developing routines. Interestingly, younger (but not older) children with AS showed poorer inhibition and a stronger tendency to exhibit repetitive response patterns. A somewhat different picture emerged on the other outcome variable of the MPT measured in the present study. In particular, no performance differences between groups were found regarding SR (independently of age), which taps the memory updating component of random sequence generation.

At the behavioral level, inflexible adherence to behavioral patterns such as restricted patterns of interests and repetitive and stereotyped behaviors are commonly observed in children with AS. While restricted, repetitive behaviors (RRBs) are a hallmark of autism spectrum disorder, the knowledge about the etiology and immediate triggers of the stereotyped behaviors is still limited. Research on potential causal origins includes theories from neurobiology and developmental psychology (for a review please see [48, 49]) that identify gene-environment neuroadaptation, lack of environmental stimulation, anxiety and arousal, as well as adaptive functions as key factors for the onset and maintenance of RRBs.

Nevertheless, cross-sectional and longitudinal studies suggest that some subtypes of restricted repetitive behaviors abate with age, especially in high-functioning individuals with autism and children with AS [59–64]. Deficient inhibition of prepotent responses may contribute to restricted, repetitive behaviors in autism (for a respective review, see [50]). Interestingly, the latter claim is supported by findings from a recent meta-analysis [52] that identified age to be a relevant moderator of inhibitory control (i.e., prepotent response inhibition) in individuals diagnosed with autism (younger individuals exhibiting poorer response inhibition).

At the brain level, neuroimaging studies disclosed that the prefrontal cortex (i.e., the left dorsolateral prefrontal cortex) is crucially involved in inhibitory processes evoked by random generation tasks (requiring participants to suppress overlearned responses or (number/letter) sequences [35, 36]. However, the latter notion is only partially corroborated by recent imaging findings [65]. While in high-functioning adult individuals with autism spectrum disorder, behavioral performance was similar to a control group in a random-motor-generation task, group differences emerged regarding brain activity in the cerebellum (known to mediate motor learning and the coordination of voluntary movements, among others, e.g., [66]). Thus, the latter findings suggest that beyond prefrontal cortices, also extra-frontal regions may mediate autism-related behavior and performance characteristics (see, for example, recent connectivity studies [67, 68] as well as respective structural brain imaging findings, e.g., [3]).

Importantly, our results of poorer inhibition of prepotent responses only in young children with AS underscore the dynamic nature of brain development in autism. There is evidence that the effective inhibition of prepotent responses requires appropriate functional connectivity among involved brain structures, which changes developmentally (e.g., [69–71]). Neuroimaging studies suggested differences in white matter maturation [72] and atypical patterns of functional connectivity observed in autism normalize over time [68], which could be the basis of age-related behavioral improvements observed in individuals diagnosed with autism. Other research, too, pointed to specific age-related changes in autism. Upon differentiating lower-level from higher-level repetitive behavior (i.e., repetitive movements vs. insistence on the maintenance of sameness, respectively; see [50]), Rinehart et al. [47] proposed that higher-level repetitive behavior becomes more evident in older children with autism. Furthermore, several authors showed that there are both typical and atypical developmental progressions of distinctive EFs in individuals with autism [52, 73, 74]. While age seems to moderate performance on prepotent response inhibition tasks, planning tasks and set-shifting tasks, no age-moderating effects were shown for spatial working memory or interference control tasks [52, 73, 74]. Hence, our finding revealing group differences solely in young children with AS regarding inhibition of prepotent responses (i.e., CR scores thought to tap low-level repetitive behavior) nicely fits the claim that specific EFs undergo age-related improvements in autistic disorders.

In the current study, we failed to find deficits in memory monitoring and updating in our study group of children and adolescents diagnosed with AS. Previous research on working memory deficits in high-functioning individuals with autism yielded inconsistent findings (for a review, see [75]), which might be mainly due to methodological differences (especially, the variety of tasks that are used to measure working memory). In most studies, working memory problems were more pronounced in complex tasks (e.g., tasks requiring manipulation instead of maintenance only) and increased when tasks imposed heavier demands on working memory load (for a review, see [75]). The SR score of the MPT task used in the current study is a more specific indicator of monitoring and updating contents in memory (i.e., tapping individuals’ capability to keep track on the chosen responses). However, because of the rather low processing requirements imposed by the MPT, also the working memory load may be considered to be relatively small, which in turn might mask subtle processing deficits related to memory monitoring/updating. Note that the finding on memory monitoring/updating was obtained with the same testing procedure as the finding on inhibition, underlining its significance.

Nonetheless, the MPT has also advantages compared to more commonly used random number generation tasks requiring participants to process (more or less) overlearned academic skills such as counting or spelling that may vary considerably between individuals. Thus, the MPT allows for a more straightforward interpretation of the inhibition and the memory components of random sequence generation [51, 57]. In random number generation (RNG), two inhibitory processes (i.e., the inhibition of overlearned responses (counting) and the permanent inhibition of developing routines) are confounded. By contrast, using unlabeled keys that are distributed over the keyboard in an irregular pattern, the CR index provided by the MPT reflects the latter component only, thus providing a purer measure of perseveration tendencies (i.e., the perseveration of sequence repetitions).

A limitation of the current study is the cross-sectional design, which limits any direct investigation of the development of specific EFs in AS. The few existing longitudinal studies examining individual differences in EFs propose that EFs might critically influence developmental trajectories of children with autism and further suggest that inter- and intraindividual EF differences could partly account for the heterogeneity in symptom severity and adaptive functioning observed in autism [16, 76]. In the current study, ten children with AS had an additional diagnosis of attention disorder treated with psychotropic medication that might have influenced individual task performance. However, boys suffering from AS with and without ADHS comorbidity did not differ in their CR or SR scores. In the current study, the two groups were well matched on age and nonverbal IQ, but not on language skills. Previously, Bishop et al. [77] suggested that inhibitory deficits in autism might be associated with poor verbal skills and inattention, rather than being specific to autism. To further examine the neurodevelopmental trajectories in AS, future research should control for structural language skills and attentional capacity in studies using random generation tests. It is important to note at this point that the motor random generation task used in the present study does not implicate verbal demands and primarily captures automatic response tendencies. Unlike many other tests of EFs, the used task does not offer any opportunity to facilitate task performance by implicit verbalization, which might influence performance depending on verbal skills (“inner speech,” see [77, 78]).

Taken together, the present study is novel as it utilized an elaborate random-motor-generation task to disentangle two components of cognitive flexibility (i.e., inhibitory processes and memory monitoring/updating) in children and adolescents with AS. Our findings disclosed poorer inhibition and more repetitive response patterns only in young children diagnosed with AS (thus reflecting age-related improvements of response inhibition in adolescents diagnosed with AS), but no such group differences in memory monitoring and updating (independently of age). These results underscore the need for a differentiated view of distinct profiles of executive (dys)functions in different age groups of children with AS. Finally, compared with clinical psychometric tests, experimental tasks such as the MPT may be better apt to disentangle more specific processes of EFs in AS.

## References

[1] American Psychiatric Association (2013). Diagnostic and statistical manual of mental disorders.

[2] Hill EL (2004). Evaluating the theory of executive dysfunctions in autism. Dev Rev.

[3] Kaufmann L, Zotter S, Pixner S, Starke M, Haberlandt E, Steinmayr-Gensluckner M, Egger K, Schocke M, Weiss EM, Marksteiner J (2013). Brief report: CANTAB performance and brain structure in pediatric patients with Asperger syndrome. J Autism Dev Disord.

[4] Ozonoff S, Pennington BF, Rogers SJ (1991). Executive function deficits in high-functioning autistic individuals: relationship to theory of mind. J Child Psychol Psychiatry.

[5] Ozonoff S, Jensen J (1999). Brief report: specific executive function profiles in three neurodevelopmental disorders. J Autism Dev Disord.

[6] Russo N, Flanagan T, Iarocci G, Berringer D, Zelazo PD, Burack JA (2007). Deconstructing executive deficits among persons with autism: implications for cognitive neuroscience. Brain Cogn.

[7] Fuster JM (2002). Frontal lobe and cognitive development. J Neurocytol.

[8] Gogtay N, Giedd JN, Lusk L, Hayashi KM, Greenstein D, Vaituzis AC, Nugent TF, Herman DH, Clasen LV, Toga AW, Rapoport J, Thompson PM (2004). Dynamic mapping ot human cortical development during childhood through early adulthood. PNAS.

[9] Kharitonova M, Martin RE, Gabrieli JD, Sheridan MA (2013). Cortical gray-matter thinning is associated with age related improvements on executive function tasks. Dev Cogn Neurosci.

[10] Selemon LD (2013). A role for synaptic plasticity in the adolescent development of executive function. Transl Psychiatry.

[11] O´Hearn K, Asato K, Ordaz M, Luna B (2008). Neurodevelopment and executivefunction in autism. Dev Psychopathol.

[12] Craig F, Margari F, Legrottaglie AR, Palumbi R, de Giambattista C, Margari L (2016). A review of executive function deficits in autism spectrum disorder and attention-deficit/hyperactivity disorder. Neuropsychiatr Dis Treat.

[13] Kleinhans N, Akshoomoff N, Delis DC (2005). Executive functions in autism and Asperger’s disorder: flexibility, fluency and inhibition. Dev Neuropsychol.

[14] Verte S, Geurts HM, Roeyers H, Oosterlaan J, Sergeant JA (2006). Executive functioning in children with an autism spectrum disorder: Can we differentiate within the spectrum?. J Autism Dev Disord.

[15] Wong D, Maybery M, Bishop DVM, Maley A, Hallmayer J (2006). Profiles of executive function in parents and siblings of individuals with autism spectrum disorders. Genes Brain Behav.

[16] Pellicano E (2012). Do autistic symptoms persist across time? Evidence of substantial change in symptomatology over a 3-year period in cognitively able children with autism. Am J Intellect Dev Disabil.

[17] American Psychiatric Association (APA) (1994). Diagnostic and statistical manual of mental disorders: DSM-IV.

[18] World Health Organization (WHO) (1992). International statistical classification of diseases and related health problems: ICD-10.

[19] Rinehart NJ, Bradshaw JL, Brereton AV, Tonge BJ (2002). A clinical and neurobehavioral review of high-functioning autism and Asperger’s disorder. Aust N Z J Psychiatry.

[20] Kaland N, Smith L, Mortensen EL (2008). Brief report: cognitive flexibility and focused attention in children and adolescents with Aspergersyndrome or high-functioning autism as measured on the computerized version of the Wisconsin card sorting test. J Autism Dev Disord.

[21] VanEylen L, Boets B, Steyaert J, Evers K, Wagemans J, Noens I (2011). Cognitive flexibility in autism spectrum disorder: Explaining the inconsistencies?. Res Autism Spect Disord.

[22] Hill E, Bird C (2006). Executive processes in Asperger syndrome: patterns of performance in a multiple case series. Neuropsychologia.

[23] Kenworthy L, Yerys BE, Anthony LG, Wallace GL (2008). Understanding executive control in autism spectrum disorders in the lab and in the real world. Neuropsychol.

[24] Ambery FZ, Russell AJ, Perry K, Morris R, Murphy DGM (2006). Neuropsychological functioning in adults with Asperger syndrome. Autism.

[25] Happe F, Booth R, Charlton R, Hughes C (2006). Executive function deficits in autism spectrum disorders and attentiondeficit/hyperactivity disorder: examining profiles across domains and ages. Brain Cogn.

[26] Liss M, Fein D, Allen D, Dunn M, Feinstein C, Morris R, Waterhouse L, Rapin I (2001). Executive functioning in high-functioning children with autism. J Child Psychol Psychiatry.

[27] Semrud-Clikeman M, Walkowiak J, Wilkinson A, Butcher B (2010). Executive functioning in children with Asperger syndrome, ADHD-combined type, ADHD-predominately inattentive type, and controls. J Autism Dev Disord.

[28] Geurts HM, Corbett B, Solomon M (2009). The paradox of cognitive flexibility in autism. Trends Cogn Sci.

[29] Leung RC, Zakzanis KK (2014). Brief report: cognitive flexibility in autism spectrum disorders: a quantitative review. J Autism Dev Disord.

[30] Miyake A, Friedman NP, Emerson MJ, Witzki AH, Howerter A, Wager T (2000). The unity and diversity of executive functions and their contributions to frontal lobe tasks: a latent variable analysis. Cogn Psychol.

[31] Huizinga M, Dolan CV, van der Molen MW (2006). Age-related change in executive function: developmental trends and a latent variable analysis. Neuropsychologia.

[32] Baddeley A (1998). Random generation and the executive control of working memory. Quart J Exp Psychol.

[33] Daniels C, Witt K, Wolff S, Jansen O, Deuschl G (2003). Rate dependency of the human cortical network subserving executive functions during generation of random number series—a functional magnetic resonance imaging study. Neurosci Lett.

[34] Geisseler O, Pflugshaupt T, Buchmann A, Bezzola L, Reuter K, Schuknecht B, Weller D, Linnebank M, Brugger P (2016). Random number generation deficits in patients with multiple sclerosis: characteristics and neural correlates. Cortex.

[35] Jahanshahi M, Dirnberger G, Fuller R, Frith CD (2000). The role of the dorsolateral prefrontal cortex in random number generation: a study with positron emission tomography. Neuroimage.

[36] Joppich G, Däuper J, Dengler R, Johannes S, Rodriguez-Fornells A, Münte TF (2004). Brain potentials index executive functions during random number generation. Neurosci Res.

[37] Knoch D, Brugger P, Regard M (2005). Suppressing versus releasing a habit: frequency-dependent effects of prefrontal transcranial magnetic stimulation. Cereb Cortex.

[38] Axmacher I, Bente D, Ferner U (1970). Informationsstatistische Untersuchungen zur Struktur einfacher Handlungsfolgen bei endogenen Psychosen [Information-statistical studies on the structure of simple response sequences in endogenous psychoses]. Arzneimittelforschung.

[39] Mittenecker E (1953). Perseveration und Persönlichkeit. 1. Teil: experimentelle Untersuchungen [Perseveration and personality, part 1: Experimental studies]. Z Exp Angew Psychol.

[40] Mittenecker E (1960). Die informationstheoretische Auswertung des Zeigeversuchs bei Psychotikern und Neurotikern. [The information-theoretical analysis of the pointing test applied in psychotics and neurotics]. Z Exp Angew Psychol.

[41] Morrens M, Hulstijn W, Lewi PJ, De Hert M, Sabbe BG (2006). Stereotypye in schizophrenia. Schizophr Res.

[42] Morrens M, Hulstijn W, Sabbe B (2006). Stereotypy in schizophrenic patients on atypical antipsychotics versus conventional neuroleptics. Eur Neuropsychopharmacol.

[43] Brown RG, Soliveri P, Jahanshahi M (1998). Executive processes in Parkinson’s disease—random number generation and response suppression. Neuropsychologia.

[44] Stoffers D, Berendse HW, Deijen JB, Wolters EC (2001). Motor perseveration is an early sign of Parkinson’s disease. Neurology.

[45] Stoffers D, Bosboom JLW, Deijen JB, Wolters EC, Berendse HW, Stam CJ (2007). Slowing of oscillatory brain activity is a stable characteristic of Parkinson’s disease without dementia. Brain.

[46] Williams MA, Moss SA, Bradshaw JL, Rinehart NJ (2002). Random number generation in autism. J Autism Dev Disord.

[47] Rinehart NJ, Bradshaw JL, Moss SA, Brereton AV, Tonge BJ (2006). Pseudo-random number generation in children with high-functioning autism and Asperger’s disorder: Further evidence for a dissociation in executive functioning?. Autism.

[48] Lopez BR, Lincoln AJ, Ozonoff S, Lai Z (2005). Examining the relationship between executive functions and restricted, repetitive symptoms of autistic disorders. J Autism Dev Disord.

[49] Leekam SR, Prior MR, Uljarevic M (2011). Restricted and repetitive behaviors in autism spectrum disorders: a review of research in the last decade. Psychol Bull.

[50] Turner M (1999). Repetitive behavior in autism: a review of psychological research. J Child Psychol Psychiatr.

[51] Schulter G, Mittenecker E, Papousek I (2010). A computer program for testing and analysing random generation behavior in normal and clinical samples: the Mittenecker pointing test (MPT). Behav Res Methods.

[52] Geurts HM, van den Bergh SF, Ruzzano L (2014). Prepotent response inhibition and interference control in autism spectrum disorders: two meta-analyses. Autism Res.

[53] Deutsches Institut für Medizinische Dokumentation und Information (DIMDI) (2014) ICD-10-GM 2014 Systematisches Verzeichnis: Internationale statistische Klassifikation der Krankheiten und verwandter Gesundheitsprobleme 11. Revision—German modification version 2014. Deutscher Ärzte-Verlag, Köln

[54] Weiss RH, Osterland J (1997). Grundintelligenztest Skala 1 (CFT1) [General Intelligence Test Scale 1].

[55] Weiss RH (2006). Grundintelligenztest Skala 2 - Revidierte Fassung (CFT 20-R) [GeneralIntelligence Test Scale 2 - Revised].

[56] Weiss EM, Fink A, Reiser EM, Schulter G, Mittenecker E, Niederstätter H, Nagl S, Parson W, Papousek I (2014). Influences of COMT and 5-HTTLPR polymorphisms on cognitive flexibility in healthy women: inhibition of prepotent responses and memory updating. PLoS ONE.

[57] Mittenecker E (1958). Die analyse “zufälliger” Reaktionsfolgen. [The analysis of “random” action sequences]. Z Exp Angew Psychol.

[58] Paulus MP, Perry W, Braff DL (1999). The nonlinear, complex sequential organization of behavior in schizophrenic patients: neurocognitive strategies and clinical correlations. Biol Psychiatry.

[59] Esbensen AJ, Seltzer MM, Lam KS, Bodfish JW (2009). Age-related differences in restricted repetitive behaviors in autism spectrum disorders. J Autism Dev Disord.

[60] Joseph L, Thurm A, Farmer C, Shumway S (2013). Repetitive behavior and restricted interests in young children with autism: comparisons with controls and stability over 2 years. Autism Res.

[61] Harrop C, McConachie H, Emsley R, Leadbitter K, Green J, PACT Consortium (2014). Restricted and repetitive behaviors in autism spectrum disorders and typical development: cross-sectional and longitudinal comparisons. J Autism Dev Disord.

[62] Howlin P, Goode S, Hutton J, Rutter M (2004). Adult outcome for children with autism. J Child Psychol Psychiatr.

[63] Seltzer MM, Krauss MW, Shattuck PT, Orsmond G, Swe A, Lord C (2003). The symptoms of autism spectrum disorders in adolescence and adulthood. J Autism Dev Disord.

[64] Shattuck PT, Seltzer MM, Greenberg JS, Orsmond GI, Bolt D, Kring S, Lounds J, Lord C (2007). Change in autism symptoms and maladaptive behaviors in adolescents and adults with an autism spectrum disorder. J Autism Dev Disord.

[65] Gilbert SJ, Bird G, Brindley R, Frith CD, Burgess PW (2008). Atypical recruitment of medial prefrontal cortex in autism spectrum disorders: an fMRI study of two executive function tasks. Neuropsychologia.

[66] Diamond A (2000). Close interrelation of motor development and cognitive development and of the cerebellum and prefrontal cortex. Child Dev.

[67] Abbott AE, Nair A, Keown CL, Datko M, Jahedi A, Fishman I, Müller RA (2016). Patterns of atypical functional connectivity and behavioral links in autism differ between default, saliency and executive networks. Cereb Cortex.

[68] Nomi JS, Uddin LQ (2015). Developmental changes in large-scale network connectivity in autism. Neuroimage Clin.

[69] Hwang K, Ghuman AS, Manoach DS, Jones SR, Luna B (2016). Frontal preparatory neural oscillations associated with cognitive control: a developmental study comparing young adults and adolescents. Neuroimage.

[70] Reineberg AE, Andrews-Hanna JR, Depue BE, Friedman NP, Banich MT (2015). Resting-state networks predict individual differences in common and specific aspects of executive function. Neuroimage.

[71] Solomon M, Yoon JH, Ragland JD, Niendam TA, Lesh TA, Fairbrother W, Carter CS (2014). The development of the neural substrates of cognitive control in adolescents with autism spectrum disorders. Biol Psychiatr.

[72] Bakhtiari R, Zürcher NR, Rogier O, Russo B, Hippolyte L, Granziera C, Araabi BN, Nili Ahmadabadi M, Hadjikhani N (2012). Differences in white matter reflect atypical developmental trajectory in autism: a tract-based spatial statistics study. Neuroimage Clin.

[73] Chen SF, Chien YL, Wu CT, Shang CY, Wu YY, Gau SS (2016). Deficits in executive functions among youths with autism spectrum disorders: an age-stratified analysis. Psychol Med.

[74] Luna B, Doll SK, Hegedus SJ, Minshew NJ, Sweeney JA (2007). Maturation of executive function in autism. Biol Psychiatr.

[75] Barendse EM, Hendriks MP, Jansen JF, Backes WH, Hofman PA, Thoonen G, Kessels RP, Aldenkamp AP (2013). Working memory deficits in high-functioning adolescents with autism spectrum disorders: neuropsychological and neuroimaging correlates. J Neurodev Disord.

[76] Pellicano E (2013). Testing the predictive power of cognitive atypicalities in autistic children: evidence from a 3-year follow-up study. Autism Res.

[77] Bishop DV, Norbury CF (2005). Executive functions in children with communication impairments, in relation to autistic symptomatology. 2: response inhibition. Autism.

[78] Russell J, Jarrold C, Hood B (1999). Two intact executive capacities in children with autism: implications for the core executive dysfunctions in the disorder. J Autism Dev Disord.

